# Toenail and blood selenium mediated regulation of thyroid dysfunction through immune cells: a mediation Mendelian randomization analysis

**DOI:** 10.3389/fnut.2024.1378969

**Published:** 2024-05-22

**Authors:** Yu-jia Jiang, Yi-quan Xiong, Tao Huang, Yun-xiao Xiao

**Affiliations:** Department of Breast and Thyroid Surgery, Union Hospital, Tongji Medical College, Huazhong University of Science and Technology, Wuhan, China

**Keywords:** selenium, mineral nutrients, antioxidant nutrients, macronutrients, immune cells, Mendelian randomization, thyroid dysfunction, diet and immunity

## Abstract

**Purpose:**

Specific nutrients found in food, such as minerals, antioxidants, and macronutrients, have a significant impact on immune function and human health. However, there is currently limited research exploring the relationship between specific nutrients, immune system function, and thyroid dysfunction commonly observed in autoimmune thyroid diseases, which manifest predominantly as hyperthyroidism or hypothyroidism. Therefore, the objective of this study was to investigate the connections between dietary traits and thyroid dysfunction, as well as the potential mediating role of immune cells, using Mendelian randomization (MR) analysis.

**Methods:**

The two-step MR analysis used single-nucleotide polymorphisms as instruments, with a threshold of *p* < 5e−08 for nutrients and thyroid dysfunction, and *p* < 5e−06 for immune cells. Data from different GWAS databases and UK Biobank were combined to analyze 8 antioxidants and 7 minerals, while the data for 4 macronutrients came from a cohort of 235,000 individuals of European. The outcome data (hypothyroidism, *N* = 3340; hyperthyroidism, *N* = 1840; free thyroxin [FT4], *N* = 49,269; thyroid-stimulating hormone [TSH], *N* = 54,288) were source from the ThyroidOmics consortium. Immune trait data, including 731 immune phenotypes, were collected from the GWAS catalog.

**Results:**

The results revealed that nutrient changes, such as lycopene, toenail and blood selenium, and α-tocopherol, impacted the immune system. Immune cells also affected thyroid function, with cDC cells promoting hypothyroidism and median fluorescence intensity (MFI) phenotypes correlating strongly with FT4 levels. Toenail and blood selenium reduce the relative cell counts (RCC) phenotypes of immune cells (CD62L− plasmacytoid DC %DC and transitional B cells %Lymphocyte), thereby diminishing its promoting effect on hypothyroidis. Furthermore, toenail and blood selenium mainly impacted phenotypes in three types of T cells (CD25 + ⁣ + CD8br, CD3 on CD45RA− CD4+, and CD45RA on Terminally Differentiated CD8br), reinforcing the negative regulation of FT4 levels.

**Conclusion:**

The role of immune cells as mediators in the relationship between nutrients and thyroid dysfunction highlights their potential as diagnostic or therapeutic markers. Toenail and blood selenium levels can indirectly impact hypothyroidism by influencing the RCC levels of two types of immune cells, and can indirectly affect FT4 levels by influencing three types of T cells.

## 1 Introduction

Thyroid dysfunction, characterized primarily by hyperthyroidism and hypothyroidism, affects millions globally (1). Autoimmune thyroid diseases (AITD), such as Hashimoto’s thyroiditis (HT), and Graves’ disease (GD), are the most common causes of thyroid dysfunction (2, 3). Recent scientific research has highlighted the significant connection between nutrition, immune function, and thyroid health. However, there remains a gap in understanding the specific relationship between individual nutrients, the immune system, and thyroid dysfunction. Further investigation in this area is warranted to optimize our understanding and management of thyroid disorders.

Understanding the epidemiology and etiology of thyroid dysfunction, involving genetic predisposition (4–7), environmental influences (8–10), is crucial for effective management. Recognized causes include smoking (11, 12), alcohol (13), drugs (14, 15), and infections (16, 17). With established correlations between nutrients and overall health, iodine is widely acknowledged as a pivotal element that exerts significant influence on thyroid health. Global iodine disparities profoundly influence thyroid dysfunction prevalence (18). However, in iodine-sufficient populations, thyroid autoimmunity, especially in HT, is often considered the primary cause. The specific causal relationship with thyroid function, apart from iodine, remains unclear. Ongoing research examines the cumulative effects of substances like chloride, thiocyanate, nitrate, and iodide found in unhealthy diets, suggesting their impact on sodium-iodide symporter-mediated radioactive iodine uptake (19). Conversely, a plant-based diet is proposed to positively influence thyroid function (20). However, comprehensive research on conventional dietary habits, macronutrients, minerals, and antioxidants in relation to thyroid dysfunction is lacking.

Apart from genetic factors, the most significant pathological mechanism underlying thyroid autoimmune diseases involves the loss of immune tolerance toward autoantigens in the thyroid gland (21). In patients with GD, HT, and postpartum thyroiditis, there is a reduction in CD8+ T cells and an increase in the CD4/CD8 ratio in peripheral blood. The presence of activated T cells expressing HLA-DR is elevated. Within the thyroid tissue, CD4+ and CD8+ T cells infiltrate and remain in an activated state. CD4+ T cells may play a dominant role in HT (22). While the circulating B cell count is normal in AITD, B cells within the thyroid tissue can produce antibodies, serving as a primary source of endogenous autoantibodies. Furthermore, cytokines and chemokines play a crucial role in the pathogenesis of autoimmune thyroiditis and GD. In the thyroid tissue, Th1 lymphocytes may stimulate the production of IFN-γ and TNF-α, triggering thyroid cells to secrete CXCL10, thereby initiating and perpetuating the autoimmune process (2). This results in the immune system attacking the thyroid and other organs, leading to thyroid dysfunction. Implicating circulating immune cells as mediators in the pathogenesis of thyroid dysfunctions. Therefore, it remains unclear whether common nutrients in the environment, including macronutrients, mineral nutrients, and antioxidant nutrients, can also affect thyroid function through specific immune cell interactions.

Mendelian randomization (MR) analysis serves as a formidable tool to evaluate the causal relationship between dietary characteristics, immune cells and thyroid dysfunction. Our objectives were to: (1) investigate the causal impact of nutrients on thyroid dysfunction; (2) determine specific immune cells as mediators in the association between nutrients and thyroid dysfunction, elucidating the proportion of mediation.

## 2 Materials and methods

### 2.1 MR analysis

**FIGURE 1:**
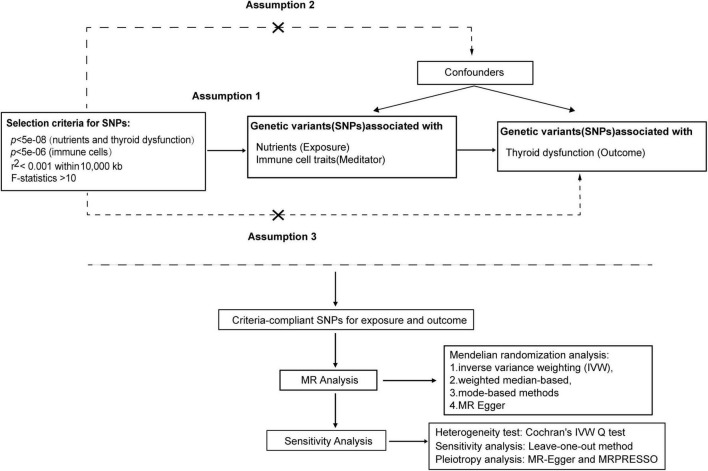
MR analysis procedure was shown in the flow chart.

The MR analysis is grounded in three key assumptions (23): (1) establishing a causal link between Single-nucleotide polymorphisms (SNPs) and the factors of exposure; (2) meticulous control for potential confounding factors influencing genetic variation, exposure, and outcome in this study; (3) affirming that genetic variation solely influences the outcome through the exposure, with no involvement of other causative factors. Our study conducted a comprehensive assessment of the causal relationship between nutrients and the risk of thyroid dysfunction via MR analysis. Furthermore, we explored the mediating effects of immune cell traits to understand their indirect influence. The flow diagram, illustrated in Figure 1, outlines the procedural steps for the MR analysis.

#### 2.1.1 The calculation method for the proportion of the mediating/indirect effect

**FIGURE 2:**
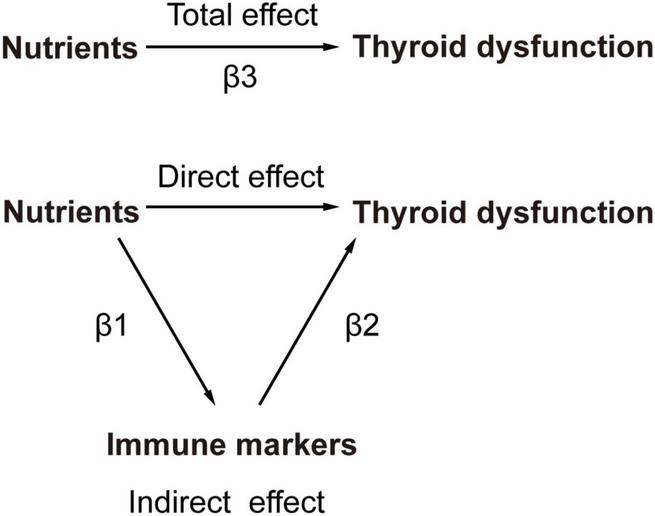
The principles of MR Analysis with mediation effects.

The calculation employed the coefficient product method to estimate the mediating effect (Figure 2). This involved determining the nutrient’s impact on circulating immune cells (β1) and then multiplying it by the effect of the immune cells on thyroid dysfunction (β2). The proportion of the mediating effect (calculated as the mediating effect divided by the total effect [(β1×β2)/β3]) was then utilized to estimate the overall proportion of the nutrient’s impact on thyroid dysfunction that is mediated through circulating immune cells (24).

### 2.2 Exposure and outcome data sources

#### 2.2.1 Thyroid dysfunction data

The genome-wide association studies (GWAS) data on thyroid diseases and thyroid function are sourced from the ThyroidOmics Consortium (25). This database includes participants of European and non-European ancestry, with exclusions for individuals who have a history of thyroid medication usage or who have undergone thyroid surgery. In each study, only subjects with TSH levels within the cohort-specific reference range were included for the TSH and FT4 analyses. TSH and FT4 were analyzed as continuous variables after inverse normal transformation. Variants with a minor allele frequency of at least 0.5% and an imputation score of at least 0.4 were included in the analysis.

This cohort includes free thyroxin (FT4) from 19 cohorts with 49,269 individuals, and thyroid-stimulating hormone (TSH) from 22 cohorts with a sample size of 54,288 individuals. It also includes two additional groups including hypothyroidism group: 3340 cases with elevated TSH levels, along with a control group of 49,983 cases within the normal reference range, and hyperthyroidism group: 1840/51,823 cases with TSH levels below the reference range. Therefore, these groups included individuals classified as subclinical hypo- or hyperthyroidism cases (25).

#### 2.2.2 Immune trait data

We sourced publicly available GWAS summary data for immune-related traits from the GWAS catalog (GCST0001391 to GCST0002121). The SardiNIA project (26) recruited 6602 volunteers aged 18–102 years (including 57% females and 43% males) from Sardinia. And 3,757 of them were immune profiled by collecting peripheral blood and then antibody-stained and processed for flow cytometry. The immune cell panel covered 731 immune phenotypes categorized into absolute cell count (ACC, *n* = 118), morphological parameters (MP, *n* = 32), median fluorescence intensity (MFI, *n* = 389), and relative cell count (RCC, *n* = 192). MP are typically used to describe the morphological characteristics of cells or tissues. On the other hand, MFI is commonly used to measure the average expression level of a particular marker in cells during flow cytometry. These phenotypes encompassed various immune cell types, such as B cells, T cells, conventional dendritic cells (cDC), myeloid cells, monocytes, TBNK cells, and the Treg panel, distributed across ACC, RCC and MFI features. The MP features included the cDC and TBNK panel. Employing a Sardinian reference panel, a comprehensive analysis was performed on 22 million SNPs.

#### 2.2.3 Nutrients data

Our study investigates three nutrient categories. The summary data for these nutrients were sourced from the total SNPs reported in published papers, which were stored in public GWAS databases and the UK Biobank databases. Antioxidant nutrients include vitamin C (abstrabe), carotene, β-carotene, lycopene, retinol, vitamin E, α-tocopherol, α-tocopherol (metabolite), and γ-tocopherol (27). The data for vitamin C (abstrabe), retinol, and vitamin E are supplemented by multiple databases as additional sources. Mineral nutrients encompass blood selenium, toenail and blood selenium, Ca, Cu, Fe, Mg, and Zn (27, 28). Macronutrients include relative intake of carbohydrates, fat, protein, and sugar, derived from the lead SNPs of the GWAS studied by Meddens et al. (29) (see Tables 1–3 for detailed information).

**TABLE 1:** Information on instrumental variables and their sources for antioxidant nutrients.

	Antioxidant nutrients^a^	First author (year)	Consortium	Sample size	Sex	Population	PMID/UKBID	IVs (nSNPs)			
								Hyper-	Hypo-	FT4	TSH
1	Vitamin C	Zheng et al. (35)	Mixed^b^	52,018	53.7% female	European	33203707	11	11	11	10
	Vitamin C (UKB)	Elsworth (63)	MRC-IEU	64,979	Mixed^c^	European	ukb-b-19390	22	23	24	24
	Ascorbate	Shine et al. (15)	TwinsUK and KORA	2,085	Mixed^c^	European	24816252	12	12	12	12
2	Carotene (UKB)	Elsworth (63)	MRC-IEU	64,979	Mixed^c^	European	ukb-b-16202	25	27	25	25
3	β-carotene	Ferrucci (64)	Mixed^b^	∼3000	Mixed^c^	European	19185284	5	5	5	5
4	Lycopene	Adamo (65)	HAPI	441	42.4% female	Caucasian	26861389	5	5	5	5
5	Retinol (UKB)	Elsworth (63)	MRC-IEU	62,991	Mixed^c^	European	ukb-b-17406	18	19	20	19
	Retinol	Mondul (66)	ATBC and PLCO	5,006	100% male	Caucasian	21878437	2	2	2	2
6	Vitamin E (UKB)	Elsworth (63)	MRC-IEU	64,979	Mixed^c^	European	ukb-b-6888	28	29	28	27
	α-tocopherol/Vitamin E	Major (67)	ATBC and PLCO	5,006	100% male	European	21729881	3	3	3	3
7	α-tocopherol (metabolite)	Shine et al. (15)	TwinsUK and KORA	7,725	Mixed^c^	European	24816252	10	10	10	10
8	γ-tocopherol	Shine et al. (15)	TwinsUK and KORA	6,226	Mixed^c^	European	24816252	13	13	13	13

^a^Multiple datasets of a certain antioxidant were used as complementary analyses to the main one.

^b^The data for this study were sourced from more than two Consortiums. The specific information can be found in the original paper.

^c^Information on the sex ratios for the whole sample were not reported or not possible to calculate.

MRC-IEU, The MRC Integrative Epidemiology Unit at the University of Bristol; NHS, The Nurses’ Health Study; HAPI, The Heredity and Phenotype Intervention Heart Study; ATBC, The Alpha-Tocopherol, Beta-Carotene Cancer Prevention Study; PLCO, The Prostate, Lung, Colorectal, and Ovarian Cancer Screening Trial; TwinsUK, The Adult UK Twins Study; KORA, The Cooperative Health Research in the Region of Augsburg; Hyper-, Hyperthyroidism; Hypo-, Hypothyroidism; UKB, UK Biobank.

**TABLE 2:** Information on instrumental variables and their sources for mineral nutrients.

	Mineral nutrients	First author (year)	Consortium	Sample size	Sex	Population	PMID	IVs (nSNPs)			
								Hyper-	Hypo-	FT4	TSH
1	Blood selenium	Evans (68)	QIMR and ALSPAC	2,603	Mixed^b^	European	23720494	13	13	13	13
2	Toenail and blood selenium	Cornelis (69)	Mixed^a^	4,162	57.4% female	European	25343990	12	12	12	12
3	Ca	O’Seaghdha (70)	Mixed^a^	39,400	Mixed^b^	Mix + European	24068962	6	6	6	6
4	Mg	Meyer (71)	CHARGE	15,366	33.9% male	European	20700443	4	4	4	4
5	Fe	Benyamin (72)	GISC	23,986	Mixed^b^	European	25352340	11	11	11	10
6	Cu	Evans (68)	QIMR	2,603	Mixed^b^	European	23720494	2	2	2	2
7	Zn	Evans (68)	QIMR	2,603	Mixed^b^	European	23720494	2	2	2	2

^a^The data for this study were sourced from more than two Consortiums. The specific information can be found in the original paper.

^b^Information on the sex ratios for the whole sample were not reported or not possible to calculate.

QIMR, The Queensland Institute of Medical Research; ALSPAC, The Avon Longitudinal Study of Parents and Children; CHARGE, The Cohorts for Heart and Aging Research in Genomic Epidemiology Consortium; GISC, The Genetics of Iron Status Consortium; Hyper-, hyperthyroidism; Hypo-, hypothyroidism.

**TABLE 3:** Information on instrumental variables and their sources for macronutrients.

	Nutrients	First author (year)	Consortium	Sex	Population	PMID	Sample size	IVs (nSNPs)			
								Hyper-	Hypo-	FT4	TSH
1	Relative intake of carbohydrate	Meddens et al. (29)	Mixed^a^	56.7% female	European	32393786	235,391	11	11	11	11
2	Relative intake of fat			60.6% female			268,922	6	6	6	6
3	Relative intake of protein						268,922	7	7	7	7
4	Relative intake of sugar						268,922	4	4	4	4

^a^The data for this study were sourced from more than two Consortiums. The specific information can be found in the original paper.

Hyper-, hyperthyroidism; Hypo-, hypothyroidism.

### 2.3 Selection of instrumental variables (IVs)

To prevent the omission of potential causal relationships, we incorporated stronger instrumental variables, allowing for an appropriate relaxation of *p*-values for immune cells. Instrumental variables were carefully selected using a threshold of *p <* 5e−08 for nutrients and thyroid dysfunction, and *p <* 5e−06 for immune cells (Using a strict *p*-value cutoff of 5e−08 led to a small number of SNPs, reducing the representativeness of instrumental variables and weakening statistical power. This threshold also excluded some immune cell variants, hindering the discovery of causal relationships. Therefore, choosing a more lenient *p*-value threshold like 5e−06 can improve the likelihood of identifying true effects while maintaining error control. This strategy enhances sensitivity, facilitating the detection of significant biological signals). And to ensure data uniformity, a clumping procedure was applied to eliminate variants showing potential linkage disequilibrium (r2 < 0.001 within 10,000 kb). Only SNPs meeting these criteria were included in our MR analysis model. To mitigate bias from weak instrumental variables, SNPs exhibiting F statistics less than 10 were removed from the analysis.

### 2.4 Statistical analysis

#### 2.4.1 Causal analysis

The methods of inverse variance weighting (IVW) (30), weighted median-based methods (31), weighted mode-based methods (32), and MR Egger (33) have been applied to causal relationship testing using the TwoSampleMR package (30). The results were mainly based on IVW (random effects), followed by sensitivity analysis. A random-effects model was applied when the corresponding *p-*value was less than 0.05. However, when the *p-*value exceeded 0.05, an IVW fixed-effect model was employed.

#### 2.4.2 Sensitivity analysis

The selected IVs underwent a heterogeneity test using Cochran’s Q statistic (33). To assess the presence of pleiotropy and confirm estimation results, we employed MR-Egger’s regression (33, 34). This approach considered an intercept term and excluded SNPs that might influence the outcome through non-exposure pathways. Additionally, the “leave-one-out” method was employed to assess the reliability of this MR results, ensuring they were not influenced by specific SNP results (35). The Causal direction verifies the directionality of each SNP using the Steiger test to ensure the prevention of reverse causality (36).

#### 2.4.3 Statistical tool

The “TwoSampleMR” package (version 0.5.7) within the R software (version 4.3.1) was utilized for causal relationship testing and sensitivity analysis. The “forestploter” package (version 1.1.1) was used for generating forest plots, visualizing the comparison of effect sizes and confidence intervals across multiple results. The code can be found in Supplementary Material 3.

## 3 Results

### 3.1 Total effect of nutrients on thyroid dysfunction

**FIGURE 3:**
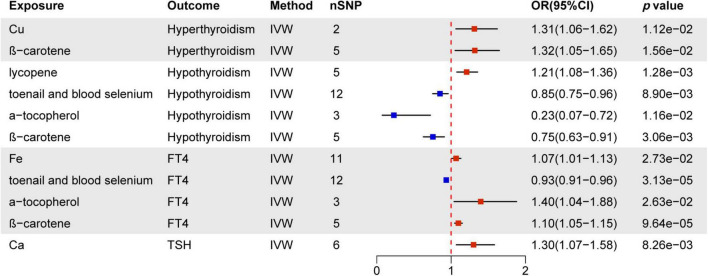
MR estimates of the causality between nutrients and thyroid dysfunction. IVW, inverse variance weighting; OR, odds ratio.

The IVW assessment reveals statistically significant associations between nutrients and thyroid dysfunction, as illustrated in Figure 3. Cu (OR = 1.31, 95% CI = 1.06–1.62, *p* = 1.12e−02) and ß-carotene (OR = 1.32, 95% CI = 1.05–1.65, *p* = 1.56e−02) are associated with an increased risk of hyperthyroidism. Lycopene increases the risk of hypothyroidism by 1.21 times, while toenail and blood selenium (OR = 0.85, 95% CI = 0.75–0.96, *p* = 8.90e−03), α-tocopherol (OR = 0.23, 95% CI = 0.07–0.72, *p* = 1.16e−02), and ß-carotene (OR = 0.75, 95% CI = 0.63–0.91, *p* = 3.06e−03) serve as protective factors against hypothyroidism. Toenail and blood selenium (OR = 0.93, 95% CI = 0.91-0.96, *p* = 3.13e−05) also exhibit a protective effect on FT4 levels. However, Fe (OR = 1.07, 95% CI = 1.01–1.13, *p* = 2.73e−02), α-tocopherol (OR = 1.40, 95% CI = 1.04–1.88, *p* = 2.63e−02), and ß-carotene (OR = 1.10, 95% CI = 1.05–1.15, *p* = 9.64e−05) pose a risk of elevated FT4 levels. Additionally, Ca increases the risk of elevated TSH by 1.30 times. Other mineral nutrients, antioxidants, and genetically predicted carbohydrates, proteins, fats, and carbohydrates are not directly related to thyroid dysfunction. Results from other methods, such as MR Egger, weighted median, and weighted mode, can be found in Supplementary Material 1 and Supplementary Tables 1–4.

### 3.2 Causal effect of immune cells on thyroid dysfunction

**FIGURE 4:**
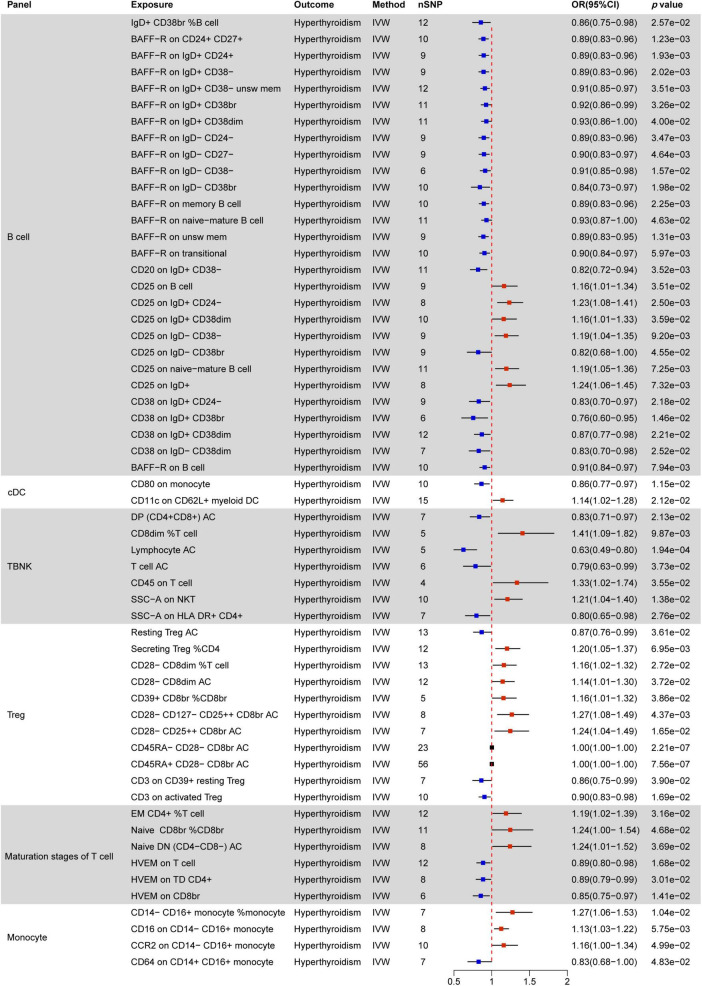
MR estimates of the causality between immune cells and hyperthyroidism. IVW, inverse variance weighting; OR, odds ratio; unsw mem, unswitched memory; AC, cell absolute count.

**FIGURE 5:**
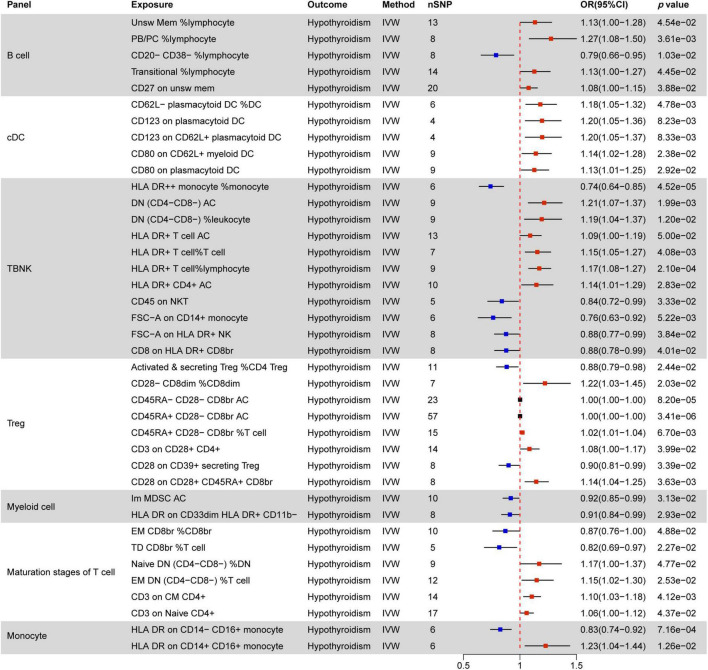
MR estimates of the causality between immune cells and hypothyroidism. IVW, inverse variance weighting; OR, odds ratio; unsw mem, unswitched memory; AC, cell absolute count; DC: Dendritic cells.

**FIGURE 6:**
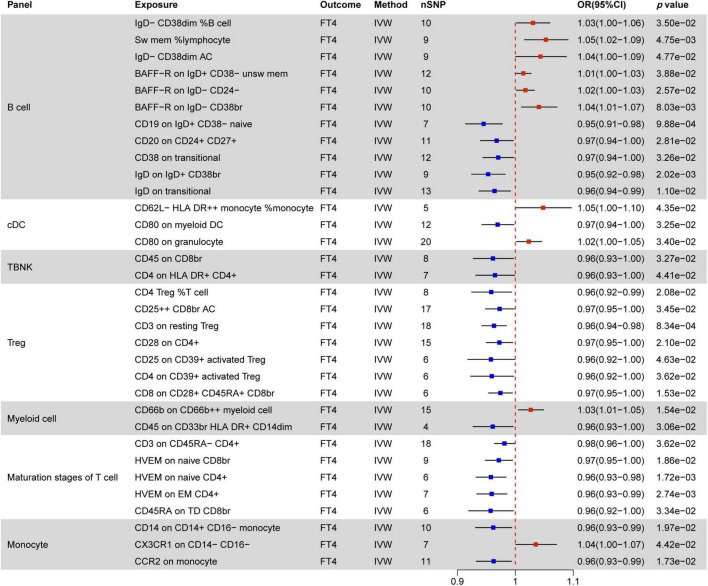
MR estimates of the causality between immune cells and FT4 level. IVW, inverse variance weighting; OR, odds ratio; unsw mem, unswitched memory; AC, cell absolute count; DC: dendritic cells.

**FIGURE 7:**
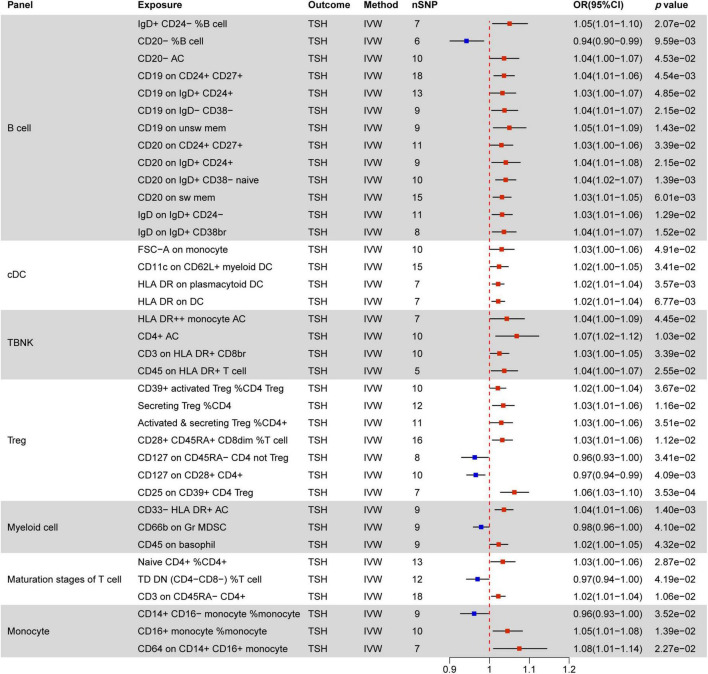
MR estimates of the causality between immune cells and TSH level. IVW, inverse variance weighting;OR, odds ratio; AC, cell absolute count; DC: dendritic cells.

Immunomodulatory effects within different immune cell types, encompassing B cells, cDCs, TBNK cells, Tregs, Myeloid cells, maturation stages of T cells, and monocytes, were analyzed in various thyroid dysfunction conditions (Supplementary Material 1 and Supplementary Tables 5–8). Figures 4–7 illustrate several immune cell factors with the potential to influence thyroid dysfunction, specifically hyperthyroidism, hypothyroidism, FT4, and TSH. Notably, all *p-*values associated with these factors are below the threshold of 0.05. Results from other methods, such as MR Egger, weighted median, and weighted mode, can be found in Supplementary Material 1 and Supplementary Tables 5–8.

#### 3.2.1 Causal effect of immune cells on hyperthyroidism

Figure 4 indicates a significant correlation between hyperthyroidism and 58 immune cell phenotypes, with B cells and T cells (TBNK, Treg, maturation stages of T cells) comprising the majority of these associations. Among B cells, the MFI phenotype exhibits the closest correlation with hyperthyroidism. Out of the 28 immune phenotypes, 27 are associated with MFI, with BRFF- being the most prevalent molecular marker (15/27), all of which act as protective factors for hyperthyroidism. The next most common markers are CD25 (7/27) and CD38 (4/27). With the exception of the CD25 on IgD− CD38br phenotype, which decreases the risk of hyperthyroidism, other phenotypes associated with the CD25 molecule increase the risk of developing hyperthyroidism. Additionally, all CD38 phenotypes are negatively correlated with hyperthyroidism.

In the combination of T cell phenotypes, the ACC phenotype is the most common. ACC phenotypes that are negatively correlated with hyperthyroidism include DP (CD4+ CD8+) AC in the TBNK panel, T cell AC, Resting Treg AC in the Treg panel, and Native DN (CD4− CD8−) AC in the maturation stages of T cells. ACC phenotypes positively correlated with hyperthyroidism all belong to the Treg panel, including CD28− CD8dim AC, CD28− CD127− CD25 + ⁣ + CD8br AC, and CD28− CD25 + ⁣ + CD8br AC. Furthermore, CD45RA− CD28− CD8br AC and CD45RA+ CD28− CD8br AC in Treg show no correlation with hyperthyroidism (OR = 1).

In TBNK cells, two SSC-A molecules related to the MP phenotype are associated with hyperthyroidism, while CD3 (2/11) in Treg and HVEM (3/6) in the maturation stages of T cells are negatively correlated with hyperthyroidism in both immune cell types.

All RCC phenotypes in the T cell combination show an increased risk of hyperthyroidism (e.g., CD8dim %T cell, Secreting Treg %CD4, CD28− CD8dim %T cell, CD39+ CD8br %CD8br), while only one RCC phenotype in B cells, IgD+ CD38br %B cell, is associated with a decreased risk of hyperthyroidism.

Only two immune phenotypes involve cDC cells, where the MFI phenotype of CD80 on monocytes plays a protective role, and CD11c on CD62L+ myeloid DC has a promoting effect. The monocyte panel includes three MFI phenotypes and one RCC phenotype associated with hyperthyroidism. Among these, CD14− CD16+ monocyte %monocyte, CD16 on CD14− CD16+ monocyte, and CCR2 on CD14− CD16+ monocyte have a promoting effect, while CD64 on CD14+ CD16+ monocyte has a protective effect.

#### 3.2.2 Causal effect of immune cells on hypothyroidism

Figure 5 illustrates associations between 39 immune phenotypes and hypothyroidism. In B cells, four RCC phenotypes and one MFI phenotype correlate with hypothyroidism. CD20− CD38− %lymphocyte acts as a protective factor, while the remaining four phenotypes (Unsw Mem %lymphocyte, PB/PC %lymphocyte, Transitional %lymphocyte, CD27 on unsw mem) serve as promoting factors.

All five immune phenotypes involving cDC cells contribute to hypothyroidism promotion. This includes CD62L− plasmacytoid DC %DC, as well as MFI phenotypes related to CD123 (2/5) and CD80 (2/5) molecules. TBNK cells often involve HLA DR molecules, where HLA DR+ T cell AC, HLA DR+ T cell %T cell, HLA DR+ T cell %lymphocyte, and HLA DR+ CD4+ AC all promote hypothyroidism, except for HLA DR + ⁣ + monocyte %monocyte and CD8 on HLA DR+ CD8br, which do not show any correlation.

In Treg cells, Activated & secreting Treg %CD4 Treg and CD28 on CD39+ secreting Treg are negatively correlated with hypothyroidism, while CD28− CD8dim %CD8dim, CD45RA+ CD28− CD8br %T cell, CD3 on CD28+ CD4+, and CD28 on CD28+ CD45RA+ CD8br show positive correlations.

Among the maturation stages of T cells, four RCC phenotypes and two MFI phenotypes are associated with hypothyroidism, with the CD3 molecule showing a positive correlation. Both immune phenotypes involving myeloid cells act as protective factors for hypothyroidism. In monocyte cells, HLA DR on CD14− CD16+ monocyte decreases the risk of hypothyroidism by 0.83 times, while HLA DR on CD14+ CD16+ monocyte increases the risk of hypothyroidism by 1.23 times.

#### 3.2.3 Causal effect of immune cells on FT4 levels

Figure 6 elucidates the impact of immune cell phenotypes on FT4 levels, serving as a direct reflection of changes in thyroid function. It is essential to recognize that FT4 levels can be influenced by various factors, such as those observed in patients with Hashimoto’s disease. These individuals may initially exhibit transient increases in FT4 levels, followed by a decline due to autoimmune attacks on the thyroid.

A total of 33 immune phenotypes are associated with variations in FT4 levels, with MFI phenotypes showing the closest relationship in 27 out of 33 instances. Notably, the majority of these immune phenotypes (23/33) act as protective factors for changes in FT4 levels. Within this context, the T cell combination emerges as pivotal, showcasing a significant negative correlation with TSH levels. All immune phenotypes in the TBNK, Treg, and Maturation stages of T cell panels (14/14) contribute to decreasing FT4 levels.

#### 3.2.4 Causal effect of immune cells on TSH levels

Figure 7 delineates 37 immune markers associated with TSH levels, with 31 of them promoting increased TSH levels. Within B cells, the most prevalent immune cell phenotypes linked to MFI markers are CD19 (4/13) and CD20 (4/13), followed by IgD (2/13). In addition to these mentioned immune cells, CD20− %B cell is associated with decreased TSH levels, while IgD+ CD24− %B cell and CD20− AC promote TSH elevation

Within cDC (FSC-A on monocyte, CD11c on CD62L+ myeloid DC, HLA DR on plasmacytoid DC, HLA DR on DC) and TBNK (HLA DR + ⁣ + monocyte AC, CD4+ AC, CD3 on HLA DR+ CD8br, CD45 on HLA DR+ T cell), all immune phenotypes are positively correlated with elevated TSH levels. The CD127 molecule, related to MFI phenotypes, shows a negative correlation with TSH levels in two distinct immune marker cells (CD127 on CD45RA− CD4 not Treg, CD127 on CD28+ CD4+). Other factors negatively correlated with TSH levels include CD66b on Gr MDSC in myeloid cells, TD DN (CD4−CD8−) %T cell in maturation stages of T cells, and CD14+ CD16− monocyte %monocyte. All other factors are positively associated with increased TSH levels.

### 3.3 Causal effect of nutrients and immune cells

**FIGURE 8:**
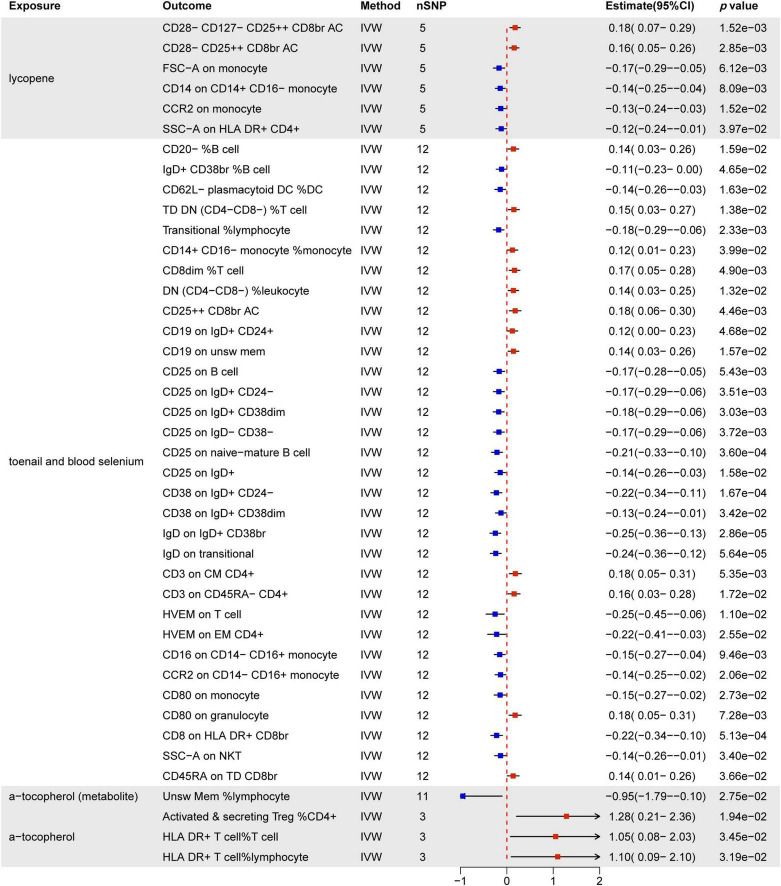
MR estimates of the causality between nutrients and immune cells. IVW, inverse variance weighting; OR, odds ratio; unsw mem, unswitched memory; AC, cell absolute count; DC: dendritic cells.

Figure 8 provides a comprehensive summary of the significant effects of nutrients on immune cells, highlighting associations with 42 immune cell phenotypes, all supported by statistically significant *p-*values. Notably, lycopene, toenail and blood selenium, and α-tocopherol emerge as influential factors.

Lycopene predominantly influences T cells and monocytes, notably by increasing CD28− CD127− CD25 + ⁣ + CD8br and CD28− CD25 + ⁣ + CD8br T cell Absolute Count. Simultaneously, it decreases FSC-A on monocytes, CD14 on CD14+ CD16− monocytes, CCR2 on monocytes, and SSC-A on HLA DR+ CD4+ T cells.

Toenail and blood selenium exhibit the most substantial impact on immune cells (32/44). This includes 15 B cell subsets, 11 T cell subsets, 4 monocyte subsets, 1 cDC cell (CD62L− plasmacytoid DC %DC), and 1 granulocyte (CD80 on granulocyte). MFI phenotypes demonstrate significant associations, with CD25 (6/32) being the most commonly expressed molecule across different immune cell types. CD38 (2/32), IgD (2/32), HVEM (2/32), CD16 (1/32), and CCR2 (1/32) molecules are negatively regulated by toenail and blood selenium across various immune cell types. Furthermore, toenail and blood selenium positively regulate immune cells associated with MFI, RCC-related phenotypes, ACC-related phenotypes, and MP-related phenotypes.

Additionally, α-tocopherol promotes ACC-related phenotypes, including activated & secreting CD4 Treg %CD4+ T cells, HLA DR+ T cells % T cells, and HLA DR+ T cells % lymphocytes. However, it inhibits the Unsw Mem %lymphocyte phenotype. Results from other methods, such as MR Egger, weighted median, and weighted mode, can be found in Supplementary Material 1Supplementary Table 9.

### 3.4 Mediation analysis

**FIGURE 9:**
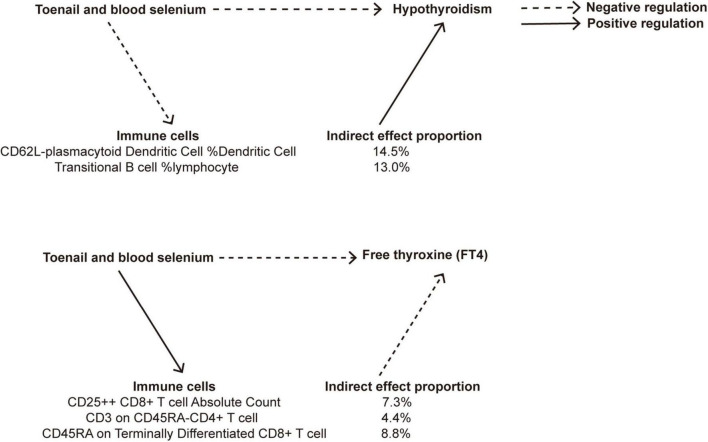
A diagram illustrating the indirect effect of nutrients on thyroid dysfunction via immune cells.

Our MR analysis revealed distinct effects of genetically predicted nutrients (Lycopene, toenail and blood selenium, and α-tocopherol and its metabolite) on immune cells. Additionally, we observed a significant association between genetically predicted immune cells and thyroid dysfunction (Table 4). Figure 9 presents the results of our two-step MR analyses, illustrating the proportion of the indirect effect mediated by immune cells in the relationship between genetically predicted nutrients and the likelihood of thyroid dysfunction.

**TABLE 4:** Information on mediation effects in MR analysis.

Exposure	Outcome	Method	nSNP	OR (95% CI)	*p* value
Toenail and blood selenium	Hypothyroidism	IVW	12	0.85 (0.75–0.96)	0.009
	FT4	IVW	12	0.93 (0.91–0.96)	0.000
Toenail and blood selenium	CD62L− plasmacytoid DC %DC	IVW	12	0.87 (0.77–0.97)	0.016
	Transitional B cell %lymphocyte	IVW	12	0.84 (0.75–0.94)	0.002
	CD25 + ⁣ + CD8+ T cell Absolute Count	IVW	12	1.20 (1.06–1.35)	0.004
	CD3 on CD45RA− CD4+ T cell	IVW	12	1.17 (1.03–1.33)	0.017
	CD45RA on Terminally Differentiated CD8+ T cell	IVW	12	1.15 (1.01–1.30)	0.037
CD62L− plasmacytoid DC %DC	Hypothyroidism	IVW	6	1.18 (1.05–1.32)	0.005
Transitional B cell %lymphocyte		IVW	14	1.13 (1.00–1.27)	0.045
CD25 + ⁣ + CD8+ T cell Absolute Count	FT4	IVW	17	0.97 (0.95–1.00)	0.035
CD3 on CD45RA− CD4+ T cell		IVW	18	0.98 (0.96–1.00)	0.036
CD45RA on Terminally Differentiated CD8+ T cell		IVW	6	0.96 (0.92–1.00)	0.033

IVW, inverse variance weighting; OR, odds ratio.

The results revealed that toenail and blood selenium levels influenced the RCC of specific immune cell, such as CD62L− plasmacytoid dendritic cells %DC and transitional B cells% lymphpcyte, mediating the association between selenium levels and hypothyroidism. Furthermore, toenail and blood selenium also impacted T cells, including one trait related to ACC: CD25 + ⁣ + CD8br T cell AC, and two traits related to MFI: CD3 on CD45RA− CD4+ T cell, and CD45RA on Terminally Differentiated CD8br T cell, The CD62L− plasmacytoid DC %DC is positively associated with hypothyroidism (OR = 1.18, 95% CI = 1.05–1.32, *p* = 4.78e−03). Toenail and blood selenium can exert a negative regulatory effect on hypothyroidism by reducing the presence of this cell type (OR = −0.14, 95% CI = −0.26 to 0.03, *p* = 1.63e−02). This finding aligns with the direct protective effect of toenail and blood selenium against hypothyroidism. The proportion of the indirect effect mediated by CD62L− plasmacytoid DC %DC was 14.5%. Similarly, the transitional B cells %Lymphocyte serves as a mediator negatively regulated by toenail and blood selenium, leading to a reduction in the risk of hypothyroidism. The proportion of the indirect effect mediated by transitional B cells %Lymphocyte is 13.0%.

CD25 + ⁣ + CD8+ T cell Absolute Count (OR = 0.97, 95% CI = 0.95–1.00, *p* = 3.45e−02), CD3 on CD45RA− CD4+ T cell (OR = 0.98, 95% CI = 0.96–1.00, *p* = 3.62e−02), and CD45RA on Terminally Differentiated CD8+ T cell (OR = 0.96, 95% CI = 0.92–1.00, *p* = 3.34e−02) are negative regulatory factors for FT4 levels. Toenail and blood selenium amplify the protective effect of three immune markers on FT4 levels, as indicated by their positive correlations (ORs of 0.18, 0.16, and 0.14, respectively). The proportion of the indirect effect mediated by these factors is 7.3%, 4.4%, and 8.8%, respectively.

### 3.5 Sensitivity analysis

The sensitivity analysis results are assessed and provided in Supplementary Material 2. The results of heterogeneity and pleiotropy tests (*p* > 0.05) suggest the absence of heterogeneity and pleiotropy in the MR study. Additionally, the “leave-one-out” analysis shows that the overall results remain relatively stable and do not change significantly after excluding each SNP. The results of the Steiger test are all “True”. All these results collectively confirm the reliable causal effect of nutrients on immune cells and thyroid function.

## 4 Discussion

Our MR analysis provides compelling evidence for the role of toenail and blood selenium in genetically reducing the risk of thyroid dyasfunction. Our study findings indicate that genetically predicted immune cells may act as mediators, and toenail and blood selenium may offer protection against the occurrence of hypothyroidism and abnormal FT4 levels.

Several nutrients are well-known for their influence on thyroid function. Iodine, an important element for thyroid hormone synthesis, is associated with hypothyroidism and goiter when deficient (37, 38). Excessive iodine can disrupt thyroid function, primarily through oxidative stress, but it generally affects only a small percentage of individuals susceptible to autoimmune thyroid diseases (39). Selenoproteins play a crucial role in regulating T cell proliferation, differentiation, and redox metabolism, reducing excessive immune responses and chronic inflammation by preventing the overproduction of reactive oxygen species (40). Zinc, functioning as a signaling molecule, antioxidant, and immune modulator (41–43), is also involved in thyroglobulin metabolism, regulating enzyme activities and modifying the structures of transcription factors related to TG synthesis (44). Zinc deficiency is implicated in hypothyroidism development (45). Additionally, copper, calcium, and magnesium impact thyroid function (46, 47).

Selenium, acting as a cofactor for enzymes in thyroid cells and participating in thyroglobulin synthesis (48), plays a vital role in governing thyroid hormone metabolism through three specific selenoproteins, namely iodothyronine deiodinases 1–3 (49). Insufficient selenium levels increases the risk of thyroid dysfunction, including autoimmune thyroid diseases, while selenium supplementation can improve clinical symptoms (50, 51), further supported by our research.

In vivo research indicates that high selenium levels promote the proliferation and differentiation of CD4 Th cells, particularly Th1 (52). Selenium supplements in aged mice enhance the proliferation of cytotoxic T cells induced by mitogens, although limited information is available regarding selenium’s impact on cytotoxic CD8 T cells (53). The deletion of the *trsp* gene, crucial for selenoprotein synthesis, influences T cell functionality and antibody secretion by B cells (54). Dietary selenium intake also influences Natural Killer (NK) cells, with serum selenium concentrations in elderly individuals positively correlated with peripheral CD16 NK cells (55). Selenium has been suggested to modulate the migration and phagocytic function of macrophages (56).

Selenium is an important risk factor for AITD (57). Selenium deficiency is often associated with immune dysfunction (58, 59). Studies suggest that Selenium may reduce thyroid antibodies by upregulating activated Treg cells (60). Selenium deficiency may upregulate Th1/Th2 effector molecules and enhance immune responses. Daily supplementation of 100 μg of Selenium has been shown to improve thyroid function and quality of life in patients by reducing interferon-γ levels and increasing interleukin-1β levels (61).

The MR study contributes genetically evidence establishing a causal relationship between nutrient intake (mineral, antioxidant nutrients, and macronutrients) and thyroid dysfunction. We found that an increase of one unit of Cu results in a 1.31-fold increase in the likelihood of hyperthyroidism. Each unit of Fe results in a 1.07-fold increase in FT4 levels, while each unit of Ca causes a 1.30-fold increase in TSH levels. β-carotene promotes hyperthyroidism and increases FT4 levels but exhibits a negative correlation with hypothyroidism. Toenail and blood selenium act as protective factors against hypothyroidism and FT4 levels. Each unit of α-tocopherol can enhance FT4 levels by 1.40 times and reduce the occurrence of hypothyroidism by 0.23 times. Furthermore, lycopene is positively associated with hypothyroidism. However, other constant nutrient intake does not have a direct causal effect on thyroid dysfunction.

Indeed, we have also discovered that a variety of immune cell phenotypes has an impact on thyroid dysfunction. Specifically, in individuals with hyperthyroidism, MFI-related phenotypes in B cells and ACC-related phenotypes in T cells are the most frequently observed. Conversely, in cases of hypothyroidism, phenotypes of all cDC cells tend to promote the occurrence of hypothyroidism. Notably, MFI phenotypes demonstrate the strongest correlation with FT4 levels. Moreover, alterations in TSH levels are associated with multiple markers within the immune system, with the majority of these markers resulting in an elevation in TSH levels.

Changes in nutrients such as lycopene, toenail and blood selenium, and α-tocopherol have distinct effects on the immune environment. For example, lycopene predominantly influences T cells and monocytes, while α-tocopherol promotes ACC-related phenotypes. However, toenail and blood selenium demonstrate the most significant impact on immune cells. Importantly, we found that the effects of nutrients on thyroid dysfunction may be mediated through alterations in the immune environment: CD62L− plasmacytoid DC %DC, and transitional B cells %Lymphocytecan serve as mediators between toenail and blood selenium levels and hypothyroidism. Toenail and blood selenium reduce the levels of these two immune cells, thereby diminishing their promoting effect on hypothyroidism. Furthermore, toenail and blood selenium enhance the levels of CD25 + ⁣ + CD8br T cell Absolute Count, CD3 on CD45RA− CD4+ T cell, and CD45RA on Terminally Differentiated CD8br T cell, thus reinforcing the negative correlation between immune cells and FT4 levels. In summary, toenail and blood selenium play a role in regulating various immune cells to counteract thyroid dysfunction. The study by Ran et al. (62) suggests that CD62L− plasmacytoid DCs have a protective effect on chronic obstructive pulmonary disease. There are currently no relevant reports on the other immune phenotypes.

Despite these findings, our study has limitations. Firstly, using SNPs as proxies for nutrient levels may have inherent shortcomings, and a more rigorous approach would involve incorporating data from Food Frequency Questionnaire surveys. Secondly, there is an overlap between abnormal FT4 and TSH levels and the definitive diagnosis of hyperthyroidism or hypothyroidism. However, not all cases of abnormal FT4 and TSH levels correspond to clinical hyperthyroidism or hypothyroidism, as some may be in a subclinical state. Nevertheless, to ensure comprehensive results, we included all available data.

## 5 Conclusion

This study establishes an association between nutrient intake and thyroid dysfunction, revealing that nutrient effects on thyroid function are mediated through immune system alterations. Toenail and blood selenium levels were identified as influential factors on specific immune cells, mediating their connection with hypothyroidism. Furthermore, toenail and blood selenium impacted other immune cell levels, reinforcing the relationship between immune cells and FT4 levels. These findings underscore the significance of selenium levels and immune modulation in understanding and addressing thyroid dysfunction.

## Data availability statement

The original contributions presented in this study are included in this article/Supplementary material, further inquiries can be directed to the corresponding authors.

## Author contributions

Y-JJ: Data curation, Formal analysis, Methodology, Software, Validation, Visualization, Writing – original draft. Y-QX: Formal analysis, Validation, Visualization, Writing – original draft. TH: Conceptualization, Project administration, Supervision, Writing – review and editing. Y-XX: Conceptualization, Data curation, Methodology, Project administration, Resources, Supervision, Writing – review and editing.
