# Supplementary material for: Toenail and blood selenium mediated regulation of thyroid dysfunction through immune cells: a mediation Mendelian randomization analysis
Source: Front Nutr. 2024 May 22;11:1378969. doi: 10.3389/fnut.2024.1378969 (PMC11150534; doi:10.3389/fnut.2024.1378969)
Supplement: Supplementary file 1 [file Data_Sheet_1.ZIP › Supplementary Materials 2/Overview of supplementary materials 2.docx]

**Sensitivity and heterogeneity analyses in supplementary materials for：**

Excel named “1”: CD3 on CD45RA- CD4+ T cell-FT4

Excel named “2”: CD25++ CD8br T cell Absolute Count-FT4

Excel named “3”: CD45RA on Terminally Differentiated CD8br T cell-FT4

Excel named “4”: CD62L− plasmacytoid Dendritic Cell %Dendritic Cell-Hypothyroidism

Excel named “5”: Toenail and blood selenium-CD3 on CD45RA- CD4+ T cell

Excel named “6”: Toenail and blood selenium-CD25++ CD8br T cell Absolute Count

Excel named “7”: Toenail and blood selenium-CD45RA on Terminally Differentiated CD8br T cell

Excel named “8”: Toenail and blood selenium-CD62L- plasmacytoid Dendritic Cell %Dendritic Cell

Excel named “9”: Toenail and blood selenium-FT4

Excel named “10”: Toenail and blood selenium-Hypothyroidism

Excel named “11”: Transitional B cell %lymphocyte-Hypothyroidism

Excel named “Sensitive analysis_All”: Comprehensive overview of all sensitivity analyses.

* The lack of analysis results for "Toenail and blood selenium-Transitional B cell % lymphocyte" is due to a limited number of SNPs available for analysis.
